# Genetic Polymorphisms in the Open Reading Frame of the CCR5 gene From HIV-1 Seronegative and Seropositive Individuals From National Capital Regions of India

**DOI:** 10.1038/s41598-019-44136-z

**Published:** 2019-05-20

**Authors:** Larance Ronsard, Vikas Sood, Ashraf S. Yousif, Janani Ramesh, Vijay Shankar, Jishnu Das, N. Sumi, Tripti Rai, Kumaravel Mohankumar, Subhashree Sridharan, Arianna Dorschel, Vishnampettai G. Ramachandran, Akhil C. Banerjea

**Affiliations:** 1 Laboratory of Virology, National Institute of Immunology, New Delhi, India; 2 Department of Microbiology, University College of Medical Sciences and Guru Teg Bahadur Hospital, Delhi, India; 3 Ragon Institute of MGH, MIT and Harvard University, 400 Technology Square, Cambridge, MA USA; 4 Renal Division, Brigham and Women’s Hospital, Harvard Medical School, Boston, MA USA; 5 Endocrinology & Toxicology Lab, Department of Zoology, University of Calicut, Kerala, India; 6 Department of Gastroenterology and Human Nutrition, All India Institute of Medical Sciences, Delhi, India; 7 Veterinary Physiology and Pharmacology, Texas A&M University, Texas, USA; 8 Department of Biochemistry and Molecular Biology, Pondicherry University, Pondicherry, India; 9 The University of St Andrews, St Andrews, KY16 9AJ UK

**Keywords:** Retrovirus, Viral epidemiology, Virus-host interactions

## Abstract

C-C chemokine receptor type 5 (CCR5) serves as a co-receptor for Human immunodeficiency virus (HIV), enabling the virus to enter human CD4 T cells and macrophages. In the absence of CCR5, HIV strains that require CCR5 (R5 or M-tropic HIV) fail to successfully initiate infection. Various natural mutations of the CCR5 gene have been reported to interfere with the HIV-CCR5 interaction, which influences the rate of AIDS progression. Genetic characterization of the CCR5 gene in individuals from the National Capital Regions (NCRs) of India revealed several natural point mutations in HIV seropositive/negative individuals. Furthermore, we identified novel frame-shifts mutations in the CCR5 gene in HIV seronegative individuals, as well as the well reported CCR5Δ32 mutation. Additionally, we observed a number of mutations present only in HIV seropositive individuals. This is the first report to describe the genetic variations of CCR5 in individuals from the NCRs of India and demonstrates the utility of investigating understudied populations to identify novel CCR5 polymorphisms.

## Introduction

UNAIDS reported that India currently has the third largest Human immunodeficiency virus (HIV) epidemic globally, with 2.1 million individuals currently infected. This burgeoning epidemic of HIV in India is linked to genetic variability and dominating emergence of various subtypes, including recombinants, and subtype B and C^1–5^, which predominantly use C-C chemokine receptor 5 (CCR5) as a co-receptor to initiate infection^6^. CCR5 interaction with HIV is critical for HIV transmission and disease progression rate^7,8^. Over the past decade, several mutations of CCR5 that seem to have a significant impact on HIV acquisition and disease progression have been identified in different populations^7,9,10^. The nature and frequency of these mutations varies between populations. Despite the large burden of HIV on the Indian population, the majority of genetic reports has focused on Caucasian, Chinese, African, and American populations^11^; only a few reports from India focus on mutations in the CCR5 gene^12^. Therefore, the purpose of this study is to characterize the genetic variations in the ORF of CCR5.

CCR5 (C-C chemokine receptor type 5) is a G-protein coupled receptor with seven transmembrane domains, that is expressed on T cells, B cells, monocytes/macrophages and microglia^13,14^ and binds the β-chemokines such as RANTES (Regulated on Activation, Normal T cell Expressed and Secreted), and MIP (Macrophages Inflammatory Proteins)-1α and -1β^15–17^. Notably, CCR5 acts as the co-receptor for R5-tropic HIV strains, and thus is crucial in initiating HIV infection^18,19^. HIV glycoprotein120 (gp120) binds to the cluster of differentiation 4 (CD4) receptor, resulting in a conformational change in gp120, which allows it to bind to either co-receptor CCR5 (R5-tropic), C-X-C chemokine receptor type 4 (CXCR4) (X4-tropic), or both (dual tropic viruses)^20–25^.

Mutations in the CCR5 gene have been shown to influence the rate of HIV progression by inhibiting co-receptor activity of CCR5 include CCR5∆32, CCR5-59653T, CCR5-59029 and CCR5-m303^18,26–29^. The CCR5Δ32 has attracted much interest as individuals carrying this mutation showed resistance to HIV infection^30,31^. The CCR5Δ32 mutation causes a frame-shift with a premature stop codon and generates a truncated form of CCR5 (215 amino acids instead of 352 amino acids in wild type CCR5). The truncated protein is not expressed on the cell surface, and thus viral binding to the receptor is hindered^31,32^. Individuals homozygous for CCR5Δ32 were protected against HIV^33,34^, and heterozygous individuals experienced delayed AIDS progression^11,35^. In 2007, a patient from Berlin was reported to be cured of AIDS after three years of treatment with bone marrow stem cell transplantation carrying homozygous CCR5∆32^36,37^, highlighting the importance of this mutation for combatting HIV infection. The frequency of the CCR5∆32 mutation was found to vary regionally, approximately 15% in Northern Europeans, 10% in Caucasians, and 19% in African Americans^18,38,39^. Amongst Asians, the frequency varies from 1 to 12%^32^. In India, the first report on heterozygosity of CCR5∆32 was documented with 1% prevalence in 1998 by our group^12^. This observation was critical for understanding genetic characteristics of Asian populations and led to further genetic screening of various mutations in the CCR5 gene.

Varying HIV transmission rate among infected individuals can mainly be attributed to polymorphisms in the co-receptors^40^. Therefore, we examined the polymorphisms in the open reading frame (ORF) of CCR5 in our population. The objective of this study was to identify the prevalence of various mutations in the CCR5 gene among NCRs of India, and to determine the genetic variations in between HIV seropositive and seronegative individuals. The CCR5 gene was amplified from peripheral blood mononuclear cells (PBMCs) of seronegative (n = 70) and seropositive (n = 72) individuals. Screening of 142 individuals in total resulted in identification of various mutations. In both seronegative and seropositive individuals, we identified (1) reported point mutations from various other population; (2) reported mutations but replaced with different amino acid at the same position, (3) novel frame-shift mutations, 32-bp (base pair) deletion mutation, and (4) novel mutations. In summary, this report describes the genetic variations occurring in the CCR5 gene from NCRs of India.

## Results

### CCR5 mutations in HIV seronegative individuals

We identified ten mutations in the ORF of CCR5 that had previously been described in various other populations^41,42^, namely K26R, L55Q, F166L, CCR5Δ32, Q194H, R223Q, Δ228K, I253T, F299S, and R319H (Table 1). These mutations were present at highly conserved amino acid positions within the CCR5 gene^9^ and most of these mutations have already been shown to be associated with delayed progression to AIDS^10,40^. Previous reports have suggested that K26R and L55Q mutations reduce expression of CCR5 for the R5 viruses, and thus alter the receptor’s functional activity. These mutations have been reported in 1.6% in Chinese and African-American individuals who are HIV seronegative^43,44^, whereas in our population, K26R and L55Q mutations were observed at 1.4% and 4.3% respectively. F166L was identified amongst Americans in the majority of HIV infected long-term non-progressive individuals^45^, and has been reported to attenuate HIV infection^46^. This mutation was observed in 1.4% of our population.

**Table 1:** Earlier reported mutations and we also observed in HIV-1 seronegative individuals.

Selected sample Code	Variations in Nucleotide	Variations in Amino acid	Nature of Variations	Allelic Frequency	Also observed in other Population
V7	A77G*	K26R*	C	(1/70)	Chinese
V150	T164A*	L55Q*	NC	(3/70)	Caucasians & African Americans
V7	T496C*	F166L*	C	(1/70)	Americans
V9, V110,	Δ554–585*	Δ32*	Δ	(2/70)	Caucasians, African Americans
V110	G582T*	Q194H*	C	(4/70)	Southern Chinese
V150	G668A*	R223Q*	NC	(2/70)	Southern Chínese
V104	A682T*	Δ228 K*	Δ	(1/70)	Caucasians
V41	T758C*	I253T*	NC	(1/70)	Southeast. Asians
V11	T896C*	F299S*	NC	(1/70)	Chinese
V97, V82	G956A*	R319H*	C	(4/70)	Caucasians

We had identified the well-known HIV protective mutation CCR5Δ32 in 1% of our population in an earlier study^12,47^. In the present study, we observed this deletion at a rate of 2.8%. Q194H alters expression of CCR5 and lies at 2% in Chinese HIV seropositive individuals^48^. In our population, this mutation was found at 5.7%. R223Q diminishes the functional activity of CCR5 but is able to bind to gp120 at lower levels^18^. The mutation had been identified in China at 4.4% and 4.1% in HIV seronegative and seropositive donors respectively^40,49^; in our population, the frequency was at 2.8%. A single lysine (K) residue deletion at the 228^th^ position in CCR5 (Δ228 K), which does not significantly alter CCR5 expression^18,40^, has previously been identified in Caucasian HIV seropositive individuals at a rate of 0.2%. This deletion was observed in 1.4% of our population. I253T alters the hydrophobicity of CCR5’s transmembrane domain but does not significantly affect receptor and co-receptor activity^49^. Both this mutation, as well as F299S, had previously been identified in 0.6% of South Chinese HIV seronegative individuals; we observed this mutation at a frequency of 1.4% in our population. R319H can alter cell surface expression of CCR5^18^. This mutation had been found in 1% of Caucasian HIV seronegative individuals. In our population, the prevalence was around 5.7%.

In our study, we observed 13 mutations at the same positions that were, however, substituted with different amino acids, and are therefore considered novel mutations in HIV seronegative individuals. This encompasses I12V, C20R, L55P, R60K, R60G, C101Y, F118L, F118S, W153L, S215P, R225P, E330K and S336G, out of which, C20R, L55P, C101Y, W153L and E330K were found slightly more frequent (Table 2). In Caucasians, I12L and C20S have been shown to alter cell surface expression, ligand binding, and co-receptor properties^18^. C20S hinders disulfide bond formation between the N-terminal and the ECL-3 (Extracellular Lumen 3), resulting in the inability to respond to chemokines by *in-vitro*^40^, as was shown with MIP-1β^18^. Both mutations occur at the N-terminal of CCR5 in 0.3% of Caucasians. We show that the Indian equivalent, I12V and C20R, occur in 1.4% and 2.8% of our population, respectively.

**Table 2:** Earlier reported mutations but substituted with different amino acids at the same positions therefore considered as novel mutations in HIV-1 seronegative individuals.

Sample Codes	Variations in Nucleotide	Variations in Amino acid	Nature of Variations	Allelic Frequency	Also observed in other Population
—	A34C	I12L	—	—	Caucasians
V59	A34G*	I12V*	C	(1/70)	NCRs of India
—	T58A	C20S	—	—	Caucasians
V6	T58C*	C20R*	NC	(2/70)	NCRs of India
—	T164A	L55Q	—	—	Caucasians
V82, V100	T164C*	L55P*	C	(4/70)	NCRs of India
—	G180T	R60S	—	—	African Americans
V112	G179A*	R60K*	C	(1/70)	NCRs of India
V150	A178G*	R60G*	NC	(1/70)	NCRs of India
—	T303A	C101X	—	—	African Americans
V6	G302A*	C101Y*	NC	(2/70)	NCRs of India
—	Δ352–354	Δ118F	—	—	Nepalese
V24	T352C*	F118L*	C	(1/70)	NCRs of India
V108	T353C*	F118S*	NC	(1/70)	NCRs of India
—	G459T	W153C	—	—	Southern Chinese
V30	G458T*	W153L*	C	(3/70)	NCRs of India
—	T644C	S215L	—	—	African Americans
S115	T643C*	S215P*	NC	(1/70)	NCRs of India
—	C673T	R225X	—	—	Africans
—	G674A	R225Q	—	—	Africans
S120	G674C*	R225P*	NC	(1/70)	NCRs of India
—	G990A	E330E	—	—	Not known
V13	G988A*	E330K*	C	(3/70)	NCRs of India
—	G1007T	S336I	—	—	Southern Chinese
V122	A1006G*	S336G*	NC	(1/70)	NCRs of India

L55Q can alter receptor activity but does not change chemokine binding affinity^18^. In Caucasians and African-Americans, the mutation has been identified in 4.1% of HIV seronegative and 0.7% of seropositive individuals. The equivalent mutation in our population, L55P, had a prevalence of 5.7%. The R60S mutation, which can alter cell surface expression, has been observed in 1.5% of African Americans^40^. The equivalent R60K and R60G mutations were found in 1.4% of our population. The N-terminal domain and three extracellular lumens of CCR5 are critical for CCR5 expression and activity. Two disulfide bonds bridge the CCR5 extracellular domains between C20-C269 and C101-C178, which ensures efficient expression of CCR5 on the cell surface^50^. The interaction with gp120 depends on ECL-2, which acts as the principal determinant of ligand selectivity^11^, and on the disulfide bonds bridging the extracellular lumens, as they are required for chemokine binding^50^. The motifs of charged and aromatic residues in the N-terminal are also crucial for the interaction between chemokines and gp120^51^. Disruption of the disulfide bond linking the N-terminal to ECL-3 strongly reduces cell surface expression of the receptor^18^. The C101X mutation in the ECL-1 of CCR5 leads to premature termination and disrupts this critical bond structure, leading to low receptor expression and impaired responsiveness to chemokines^18^. The mutation had previously been observed in 1.4% of HIV-negative African-Americans. In our population, the equivalent mutation C101Y appeared in 2.8% of individuals.

A previously identified mutation, a deletion of phenylalanine (F) at the 118^th^ amino acid position in the third transmembrane domain of CCR5, results in attenuated AIDS progression in the Nepalese population^52^. We observed the mutations F118L and F118S at the same locations in our population, with a frequency of 1.4%. W153C, a mutation which can alter expression of CCR5 on the cell surface^53,54^, had been reported in HIV seronegative individuals. We observed the location-equivalent W153L mutation in 4.3% of our population. S215L, a mutation of the fifth transmembrane of CCR5 which results in the alteration of CCR5 protein expression on the cell surface, and consequently low binding affinity towards MIP-1 β^40^, has been found in 1% of African American HIV seronegative individuals^27^. In our population, the equivalent S215P mutation was identified in 1.4%.

The R225Q mutation results in premature termination of translation and leads to the receptors inability to be expressed on the cell surface, bind chemokines, and respond to HIV infection^55^. This mutation has been found in 0.7% of African seronegative individuals^56^. The presence of R225P was observed at 1.4% in our present report. E330E has been identified in the C-terminal of CCR5 in HIV infected long-term non-progressive patients of African descent^18^, suggesting an inhibitory effect. In our population, E330K was observed at 4.3%. S336I has been found in the C-terminal of CCR5 in 0.4% of HIV seropositive individuals from Southern China^53,54^. The equivalent S336G mutation was observed at 1.4% in our population.

Sixteen novel mutations were identified in HIV seronegative individuals namely S17P, V25A, S38L, L50P, V51A, L77P, L81P, F85L, F112L, F158L, F158V, H181S, I198V, I212V, F311S and G344R. Amongst these, S38L, L50P, F85L, F158L, H181S and F311S appeared slightly more frequent than other novel mutations (Table 3). Various frame-shifts, either due to insertion or deletion of a nucleotide, were identified. Insertion of Guanidine (G) at the 467^th^ nucleotide position results in a frame-shift at the 156^th^ amino acid position of CCR5, which leads to a truncated form CCR5 with 226 amino acids instead of 352 (wild type CCR5). This frame-shift was observed in 5.7% of our population. Deletion of Thymine (T) at the 498^th^ nucleotide position results in a frame-shift at the 166^th^ amino acid position of CCR5, which leads to a truncated protein of 227 amino acids, which we found in 2.8% of our population. Deletion of Adenine (A) at the 658^th^ nucleotide position results in a frame-shift at the 220^th^ position of amino acid, which results in a truncated protein of 233 amino acids. This was observed in 5.7% of NCRs of India (Table 4).

**Table 3:** Novel mutations in HIV-1 seronegative individuals.

Sample Codes	Variations in Nucleotide	Variations in Amino acid	Nature of Variations	Allelic Frequency
V101, V21	T49C*	S17P*	NC	(2/70)
V59, V108	T74C*	V25A*	C	(2/70)
V97, V82, S115	C113T*	S38L*	NC	(4/70)
V68, V100	T149C*	L50P*	C	(3/70)
V12, V32	T152C*	V51A*	C	(2/70)
V21, V95	T230C*	L77P*	C	(2/70)
V122, V32	T242C*	L81P*	C	(2/70)
V97, V82	T253C*	F85L*	C	(3/70)
V21, V24	T334C*	F112L*	C	(2/70)
V68, V110	T474A*	F158L*	C	(2/70)
V44, V77	T472G*	F158V*	C	(2/70)
V16, V26, V82	T543A*	H181S*	C	(4/70)
S82.1, S82.2	A592G*	I198V*	C	(2/70)
V59, V43	A634G*	I212V*	C	(2/70)
V97, V82	T932C*	F311S*	NC	(3/70)
V5, V12	G1030A*	G344R*	NC	(2/70)

**Table 4:** Novel frame-shifts in HIV-1 seronegative individuals.

Sample Codes	Variations in Nucleotide	Variations in Amino acid	Wild/mutant	Causes	Frequency
V44, V68, V77, V82	Insert 467G*	FS at 156*	352/226	Pre-mature end	(4/70)
V1, V23	Δ498T*	FS at 166*	352/227	Pre-mature end	(2/70)
V11, V21, V59, V82	Δ658A*	FS at 220*	352/233	Pre-mature end	(4/70)

### CCR5 mutations in HIV seropositive individuals

The single mutation F107L, previously reported in African populations^9^, results in normal CCR5 expression, chemokine binding and co-receptor properties by *in-vitro*^55^. In our population, this mutation was identified at a higher percentage of 8.3% (Table 5). The five mutations A29T, A73P, W86G, C101R and W153R had previously been reported in different populations (Table 6). In our population, we observed mutations at the same positions that were substituted with different amino acids and are therefore considered novel mutations. A29S results in normal expression of CCR5 on the cell surface, thus enabling HIV to cause infection^40^. This mutation had been found in 1.5% of African-Americans. We identified the position-equivalent A29T mutation in 2.7% of our population. The A73V mutation triggers HIV infection and has been shown to increase ligand binding affinity by 4-8-fold *in-vitro*^18^. We observed the position-equivalent A73P mutation in 5.5% of our population. W86C has been found in 1.4% of African-American HIV infected individuals^9^, while W86G was found at the elevated rate of 8.3% in our population.

**Table 5:** Earlier reported mutations and we also observed in HIV-1 seropositive individuals.

Sample Codes	Variations in Nucleotide	Variations in Amino acid	Nature of Variations	Allelic Frequency	Also observed in other Population
21, 59, 56, 63, 62, 65	C319T*	F107L*	NC	(6/72)	Africans

**Table 6:** Earlier reported mutations but substituted with different amino acids at the same positions therefore considered as novel mutations in HIV-1 seropositive individuals.

Sample Codes	Variations in Nucleotide	Variations in Amino acid	Nature of Variations	Allelic Frequency	Also observed in other Population
—	G85T	A29S	—	—	African Americans
N1, N4	G85A*	A29T*	C	(2/72)	NCRs of India
—	G218T	A73V	—	—	Caucasians
56, 63, 62, 65	G217C*	A73P*	NC	(4/72	NCRs of India
—	G258C	W86C	—	—	South Africans
21, 59, 56, 63, 62, 65	T256G*	W86G*	NC	(6/72)	NCRs of India
—	T303A	C101X	—	—	African Americans
E73	T301C*	C101R*	NC	(1/72)	NCRs of India
—	G459T	W153C	—	—	Southern Chinese
N3, 5795G, 5611D, 6159C	C457T*	W153R*	C	(4/72)	NCRs of India

C101X, a mutation of the ECL-1, results in premature termination, and consequently lowered cell surface expression, as well as altered chemokine binding affinity. C101X has been identified in 1.4% of African-American HIV negative individual. The position-equivalent C101R mutation was found in the same percentage of 1.4 in our population. W153C has been observed in HIV infected patients of the southern regions of China^57^ while the position-equivalent W153R was observed in our population at 5.5%. Nine novel mutations namely R31G, F41L, A87V, H88R, A90P, G97E, T177I, R235W and T282A were observed in HIV infected individuals of our population. F41L, H88R, and G97E were observed in 4.5%, and A90P was present at an elevated rate of 6.9% (Table 7).

**Table 7:** Novel mutations in HIV-1 seropositive individuals.

Sample Codes	Variations in Nucleotide	Variations in Amino acid	Nature of Variations	Allelic Frequency
62, 65	C91G*	R31G*	C	(2/72)
N3, 5795G, 5611D, 6159C	T121C*	F41L*	NC	(4/72)
62, 65	C260T*	A87V*	NC	(2/72)
N3, 5795G, 5611D, 6159C	A263G*	R88H*	NC	(4/72)
21, 59, 56, 63, 65	G268C*	A90P*	NC	(5/72)
56, 63, 62, 65	G290A*	G97E*	C	(4/72)
N1, N4	C530T*	T177I*	C	(2/72)
N1, N4	A703T*	R235W*	C	(2/72)
N1, N4	A844G*	T282A*	C	(2/72)

## Discussion

Over the years, clinical trials have been conducted in different locations with the aim of strengthening cellular immune response to protect against HIV; however there has been no definitive cure^58^. This speaks for the complex nature of HIV, which is characterized by genetic diversity, high mutation rates, and issues of antiretroviral drug toxicity and viral resistance, presenting further obstacles. Following the breakthrough discovery of CCR5Δ32 as a protective mutant against HIV, research has shifted its focus towards targeting of the CCR5 co-receptor^59,60^. In this report, we have identified several novel mutations in seronegative and seropositive individuals from the NCRs of India and compared their presence to other populations.

In seronegative individuals, we identified several mutations that had also been reported in different populations. K26R, F166L, Δ228K, I253T and F299S were identified in 1 out of 70 individuals. CCR5Δ32 and R223Q were identified in 2 out of 70 individuals. L55Q was observed in 3 out of 70 individuals. Q194H and R319H was found in 4 out of 70 individuals. It is important note that these mutations (K26R, C178R, Q194H and R223Q) were also observed in the Chinese population^52,61^. In a 1998 study, CCR5Δ32 was a rare mutation in India expressed in about 1% of the population^12^, whereas in the present study, we observed this deletion at a percentage of 2.8. The increase in the frequency of delta 32 mutation in CCR5 among NCRs of India may be attributed to multiple factors. One relevant factor is India’s rapid population growth, with this mutation increasing by natural selection, as prevalence of this mutation aids survival against HIV.

In addition to CCR5Δ32, nine other mutations namely K26R, L55Q, F166L, Q194H, R223Q, Δ228K, I253T, F299S, and R319H (Table 1) had previously been identified in African-American, Hispanic, Chinese, Israeli and Japanese populations^27,57,62,63^. We also observed those mutations in our population, however at different rates^11,64^. The differences in allelic percentage of these mutations between populations may reflect the reason for the varying risk for HIV infection.

We further identified a class of mutations at the same amino acid positions as mutations that had been identified in other populations. However, as they were substituted with different amino acids, they could be classed as novel mutations. The thirteen mutations include I12V, R60K, R60G, F118L, F118S, S215P, R225P and S336G, which only occurred in 1 out of 70 individuals; C20R and C101Y were observed in 2 out of 70 individuals, and W153L as well as E330K were found in 3 out of 70 individuals. L55P was identified in 4 out of 70 individuals. Most of these mutations were common among Caucasians, Africans and Chinese^18^; our study constitutes the first report of these mutations in NCRs of India.

Apart from these mutations, 16 novel mutations were observed for the first time in our chosen population such as S17P, V25A, S38L, L50P, V51A, L77P, L81P, F85L, F112L, F158L, F158V, H181S, I198V, I212V, F311S and G344R. Amongst these, L50P, F85L and F311S were found in 3 out of 70 individuals. S38L and H181S mutations were identified in 4 out of 70 individuals.

Novel frame-shifts due to singe nucleotide insertions or deletions were identified at amino acid positions 156, 166, and 220. These mutations resulted in frame-shifts at 467, 498, 658 nucleotide positions respectively, resulting in premature termination of translation. As a result, the gene product of CCR5 lacked approximately 118 to 126 amino acid residues in the C-terminal cytoplasmic tail, i.e. loss of three transmembrane domains, thus reducing its length from 352 amino acids, as found in wild-type CCR5, to a truncated form of 226–233 amino acids (Table 4). Frame-shifts at amino acid positions 156 and 220 were found at a higher percentage, in 4 out of 70 individuals. The C-terminal transmembrane domains are essential for gp120 interaction, and any changes in these regions could affect initial HIV entry^31^. Our results are the first report identifying these frame-shifts amongst our population; however, the functional consequences of these mutations are yet to be studied.

In seropositive individuals, F107L had been reported in Africans. In our population, we observed this mutation in 6 out of 72 individuals. Novel mutations located at earlier reported amino acid positions but substituted with different amino acids, were reported in our population. The five mutations include A29T, A73P, W86G, C101R, and W153R, out of which W86G was observed six times and W153R four times in our group of 72 participants. Nine novel mutations were identified in HIV seropositive individuals. The five mutations R31G, A87V, T177I, R235W and T282A were identified in 2 out of 72 individuals; the three mutations F41L, R88H and G97E were identified in 4 out of 72 individuals; and A90P was identified in 5 individuals out of 72.

We observed certain mutations more frequently than others, namely F41L, A73P, W86G, R88H, A90P, G97E, F107L and W153R in HIV seropositive individuals. We noticed that the number of mutations in CCR5 was fewer in individuals undergoing ART, when compared to individuals on non-ART. Interestingly, we didn’t find any truncated forms of CCR5 in HIV seropositive individuals, which suggests that presence of the full frame of CCR5 is essential for successful HIV infection.

In our cohort study, genotypic frequencies of the majority of mutants did not significantly differ between HIV seropositive and seronegative individuals (see Supplemental Table S2). However, certain mutations, namely S38L, L55P, H181S, Q194H and R319H including the frame shifts at 156 and 220 were enriched in HIV seronegative individuals. At the same time, mutations such as F41L, A73P, W86G, R88H, A90P, G97E, F107L and W153R were enriched in HIV seropositive individuals (Table 8). These findings are congruent with other reports describing some of these mutations, in which the association between CCR5 genotypes and HIV progression rate was reported^10,18^; however, most of the identified novel mutations are yet to be characterized in relation to their functional consequences.

**Table 8:** Comparison of mutations between HIV-1 seronegative and seropositive individuals.

Mutations	HIV-1 negative		HIV-1 positive		p-value (Chi-square test)	q-value (post Benjamini-Hochberg multiple testing correction)
	Total number of individuals carrying the mutation	Total individuals (n)	Total number of individuals carrying the mutation	Total individuals (n)		
S38L*	4	70	0	72	0.0396	0.1727
L55P*	4	70	0	72	0.0396	0.1727
H181S*	4	70	0	72	0.0396	0.1727
Q194H*	4	70	0	72	0.0396	0.1727
R319H*	4	70	0	72	0.0396	0.1727
FS at 156*	4	70	0	72	0.0396	0.1727
FS at 220*	4	70	0	72	0.0396	0.1727
F41L*	0	70	4	72	0.0396	0.1727
A73P*	0	70	4	72	0.0396	0.1727
W86G*	0	70	6	72	0.0136	0.1727
R88H*	0	70	4	72	0.0396	0.1727
A90P*	0	70	5	72	0.0248	0.1727
G97E*	0	70	4	72	0.0396	0.1727
F107L*	0	70	6	72	0.0136	0.1727
W153R*	0	70	4	72	0.0396	0.1727

Abbreviations:

C-Conserved; NC- Non-Conserved;

NR- Not Reported FS-Frame-shift;

*-Found in our study; X-Stop Codon;

Δ-Deletion;Insert-Insertion.

Available methods of therapeutic interventions have failed to fully cure HIV infection due to partial restoration of the immune system and continuous progression to AIDS, which warrants the necessity to develop novel strategies. This study presents a report of genetic variations occurring in the ORF of CCR5 in HIV seronegative and seropositive individuals. As a next step, the functional implications (effects on CCR5 expression on the cell surface and the role of identified mutations in HIV progression) of these natural mutations will be studied in detail. Modification of CCR5 cell surface expression through natural mutations using novel techniques such as siRNA silencing, zinc-finger nuclease silencing, CRISPR-cas9 system, and gene therapy^65–70^ may presents a novel approach to the development of chemokine-based therapeutics against HIV.

## Materials and Methods

### Ethics statement

This study was approved by Research Project Advisory Committee, Institutional Biosafety Committee and Institutional Ethical Committee from Human Research of University College of Medical Sciences and GTB hospital, Delhi, India and from PGIMER, Chandigarh, India. Ethics committees of each of these institutes independently approved the written informed consents which were obtained from HIV seropositive individuals and from the guardians of HIV seropositive children participants involved in this study. All the experiments were performed in accordance with relevant guidelines and regulations.

### Patient selection and ethics statement

HIV seronegative (n = 70) and HIV seropositive (n = 72) samples chosen for the study were collected from NCRs of India (Haryana, Punjab, Chandigarh, Delhi, Uttar Pradesh) from patients who were registered and monitored at the ART clinic of GTB hospital, Delhi and PGIMER, Chandigarh within the period from 2005 to 2013. The participants were made up of 45% males, 13% HIV mother to child pairs, and 42% other females (see Supplemental Table S1).

### Genomic DNA isolation and PCR for CCR5 genes

Genomic DNA was extracted from PBMCs of HIV seropositive individuals by QIAamp DNA Blood Mini Kit (Qiagen) and sequence spanning the open reading frame (ORF) of CCR5 was PCR amplified using the following primers.

FP: 5′-GGG TGG AAC AAG ATG GAT TAT CAA GTG-3′

RP: 5′-ACC CCC AGC CCA GGC TGT GTA T-3′

PCR amplification of CCR5 genes was carried out in 15 μl reaction volumes in separate PCR tubes. The reaction mixture contained 400 ng genomic DNA (3 µl), 10X PCR Buffer (1.5 µl) 10 mM dNTP mix (0.37 µl), 25 pmol of each primer, 1.5 U Taq DNA polymerase (Takara) and DNase RNase Nuclease free water (7.93 µl). The reaction mixture was subjected to 94 °C for 5 mins as initial denaturation, followed by 40 cycles at 94 °C for 1 min, 70 °C for 30 sec and 72 °C for 1 min, and a final extension step was carried out at 72 °C for 7 mins. The PCR product was then resolved on 1.5% agarose gel after electrophoresis. The amplicons were eluted out from the gel by using Qiagen Gel extraction kit (Qiagen).

### CCR5 genotyping by cloning and sequencing

The gel purified PCR products (see Supplemental Fig. S1) were cloned in pGEM-T Easy vector system (Promega). The ligation reaction was incubated at 4 °C for 10 hrs, and the ligation mix was plated on LB ampicillin plates with *E.coli* DH5α strain as host. The plates were then incubated overnight at 37 °C. The positive clones were selected by picking a single colony, grown in 5 ml LB Broth with ampicillin antibiotic (100 µg/ml), and incubated overnight at 37 °C. Plasmid DNA was isolated from the culture by QIAprep Spin Mini Kit (Qiagen). The positive clones were screened by restriction digestion of plasmid DNA with EcoRI in a 10 μl reaction volume at 37 °C for 2 hrs. The digested products were analyzed on a 1.5% agarose gel after electrophoresis and the amplified bands were screened for positive clones by restriction digestion of the products with EcoR1 (see Supplemental Fig. S2). Three clones from each individual were subjected to sequencing from LabIndia and SciGenom laboratories by dideoxy chain termination method. CCR5 open reading frame (ORF) was then translated to amino acids by Gene Runner and the amino acid sequences were aligned with reference sequence (NM_000579) by ClustalW to identify novel mutants.

### Statistical analysis

In this study, Chi-square test was used to assess the statistical significance of the mutations between the two groups using GraphPad Prism8 and the values p < 0.05 was considered to be statistically significant. Multiple comparison was tested using the Benjamini-Hochberg test for the mutations between the two groups and the values q < 0.2 was considered to be statistically significant.

### Accession numbers

CCR5 sequences (n = 110) from NCRs of India [GenBank KM355846 - KM355955].

## Supplementary information

Supplemental tables and figures
