# Supplementary material for: Genetic Polymorphisms in the Open Reading Frame of the CCR5 gene From HIV-1 Seronegative and Seropositive Individuals From National Capital Regions of India
Source: Sci Rep. 2019 May 20;9:7594. doi: 10.1038/s41598-019-44136-z (PMC6527560; doi:10.1038/s41598-019-44136-z)
Supplement: Supplementary file 1 — Supplemental tables and figures [file 41598_2019_44136_MOESM1_ESM.docx]

Supplemental files:

**Genetic Polymorphisms in the Open Reading Frame of the CCR5 gene From HIV-1 Seronegative and Seropositive Individuals From National Capital Regions of India**

Larance Ronsard^1,2,3*^, Vikas Sood^1,2^, Ashraf S. Yousif^3^, Janani Ramesh^4^, Vijay Shankar^1^, Jishnu Das^3^, Sumi N^5^, Tripti Rai^6^, Kumaravel Mohankumar^7^, Subhashree Sridharan^8^, Arianna Dorschel^9^, Vishnampettai G Ramachandran^2^ and Akhil C Banerjea^1*^

**Authors’ affiliation**

^1^Laboratory of Virology, National Institute of Immunology, New Delhi, India.

^2^Department of Microbiology, University College of Medical Sciences and Guru Teg Bahadur Hospital, Delhi, India.

^3^Ragon Institute of MGH, MIT and Harvard University, 400 Technology Square, Cambridge, MA, USA.

^4^ Renal Division, Brigham and Women’s Hospital, Harvard Medical School, Boston, MA, USA.

^5^Endocrinology & Toxicology Lab, Department of Zoology, University of Calicut, Kerala, India.

^6^Department of Gastroenterology and Human Nutrition, All India Institute of Medical Sciences, Delhi, India.

^7^Veterinary Physiology and Pharmacology, Texas A&M University, Texas, USA.

^8^Department of Biochemistry and Molecular Biology, Pondicherry University, Pondicherry, India.

^9^The University of St Andrews, St Andrews KY16 9AJ, UK.

***Correspondence authors**

Dr. Akhil C. Banerjea, Chief Staff-Scientist VII, Laboratory of Virology, National Institute of Immunology, Aruna Asaf Ali Marg, New Delhi-110067, India; Tel No: +91-011-26703616; Fax No: +91-011-26742125; Email IDs: [akhil@nii.res.in](mailto:akhil@nii.res.in), [akhil@nii.ac.in](mailto:akhil@nii.ac.in).

Dr. Larance Ronsard, Ragon Institute of MGH, MIT and Harvard University, 400 Technology Square, Cambridge, MA 02139, USA Email IDs: [LRonsard@mgh.harvard.edu](mailto:LRonsard@mgh.harvard.edu) and [LRonsard@broadinstitute.org](mailto:LRonsard@broadinstitute.org), Mob: +1 857-770-8126.

**Running title:** Genetic variations in the open reading frame of CCR5 gene.

**Keywords:** HIV-1, AIDS, CCR5, CCR5Δ32, Resistant mutants.


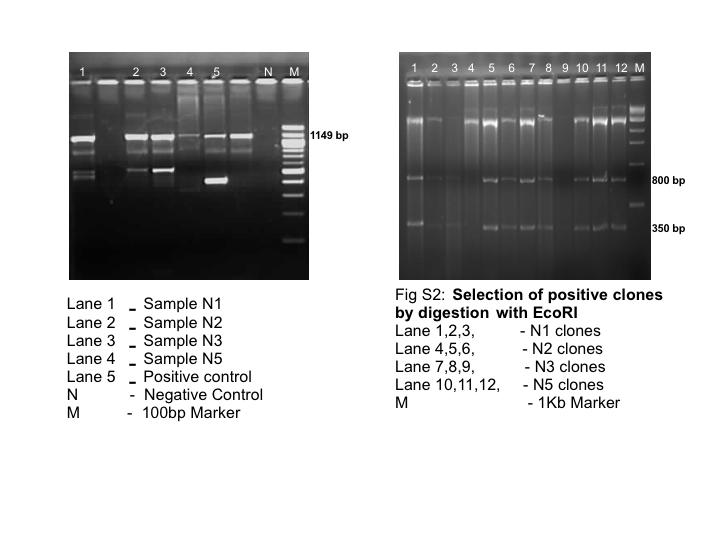


Figure S1. **PCR amplification of CCR5 from samples.** The figure shows the PCR product of 1149 bp. Lane 1 was sample N1, Lane 2 was sample N2, Lane 3 was sample N3, and Lane 4 was sample N5, Lane 5 is positive control (previously sequenced confirmed CCR5 clone), Lane N is negative control (water), Lane M is 100bp ladder. The N1, N2, N3 and N5 samples were from HIV-1 seropositive individuals.


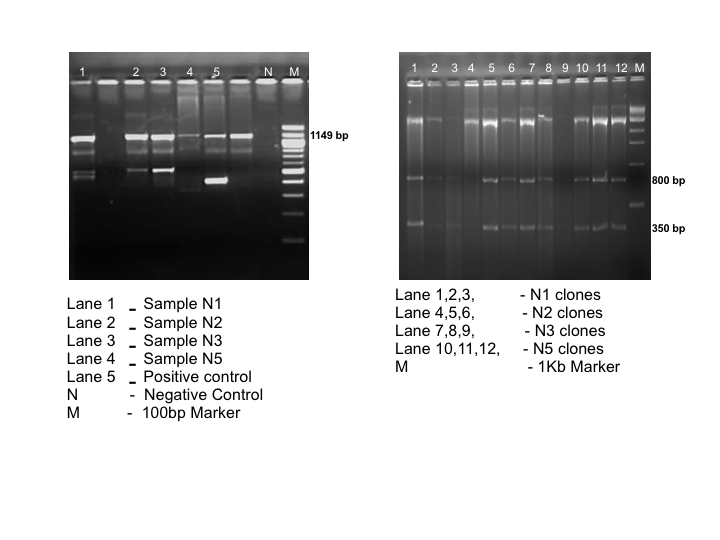


Figure S2. **Selection of positive clones by digestion with EcoRI.** The figure shows restriction digestion of the 1149bp clone yielded two bands at 800bp and 349bp. The two bands are due to the presence of one internal EcoR1 restriction site. Lane 1 to 3 were cloneN1, Lane 4 to 6 were cloneN2, Lane 7 to 9 were cloneN3, Lane 10 to 12 were cloneN5, and Lane M is 1kb ladder. The N1, N2, N3 and N5 samples were from HIV-1 seropositive individuals.

|  |  |  |  |  |  |  |
| --- | --- | --- | --- | --- | --- | --- |
| Code No | Age | Sex | Positivity known since | Possible Route of transmission | ART status | CD4 |
| N1 | 32 | M | 2007 | heterosexual | ART –ve | 169 |
| N2 | 40 | M | 2007 | heterosexual | ART +ve | 62 |
| N3 | 34 | M | 2008 | heterosexual | ART +ve | 114 |
| N4 | 45 | M | 2008 | heterosexual | ART –ve | 37 |
| N5 | 54 | M | 2009 | heterosexual | ART +ve | 60 |
| 56 | 30 | M | 2007 | heterosexual | ART –ve | 136 |
| 63 | 23 | M | 2007 | heterosexual | ART –ve | 458 |
| 62 | 25 | M | 2009 | heterosexual | ART –ve | 82 |
| 65 | 40 | M | 2008 | heterosexual | ART –ve | 69 |
| 21 | 27 | M | 2008 | heterosexual | ART –ve | 649 |
| 59 | 28 | F | 2006 | heterosexual | ART –ve | 252 |
| 74 | 20 | M | 2009 | heterosexual | ART +ve | 412 |
| 72 | 28 | M | 2008 | heterosexual | ART +ve | 153 |
| 5611D | 23 | F | 2008 | heterosexual | ART –ve | 43 |
| 71 | 29 | M | 2008 | heterosexual | ART +ve | 111 |
| 6159C | 36 | M | 2008 | heterosexual | ART –ve | 345 |
| D70 | 30 | F | 2008 | heterosexual | ART –ve | 419 |
| 9 | 30 | F | 2008 | heterosexual | ART +ve | 497 |
| E73 | 9 | F | 2008 | vertical | ART +ve | 403 |
| E71 | 6 | F | 2008 | vertical | ART –ve | 972 |
| S1 | 33 | M | 2005 | Heterosexual | ART –ve | 364 |
| S2 | 37 | M | 2006 | Heterosexual | ART –ve | NA |
| S3 | 35 | F | 2004 | Heterosexual | ART +ve | 253 |
| S4 | 23 | F | 2007 | Heterosexual | ART –ve | NA |
| S5 | 29 | M | 2008 | Heterosexual | ART +ve | 111 |
| S6 | 36 | M | 2008 | Heterosexual | ART –ve | 345 |
| S17 | 38 | M | 2008 | Heterosexual | ART +ve | 234 |
| S19 | 27 | F | 2007 | Heterosexual | ART +ve | 320 |
| S57 | 36 | M | 2007 | Heterosexual | ART +ve | 354 |
| S80 | 24 | F | 2008 | Heterosexual | ART –ve | 481 |
| S81 | 34 | M | 2008 | Heterosexual | ART +ve | 211 |
| VT1 | 24 | F | 2007 | Heterosexual | ART +ve | 152 |
| VT2 | 4 | M | 2007 | Vertical | ART +ve | 727 |
| VT3 | 30 | F | 2008 | Heterosexual | ART –ve | 233 |
| VT4 | 8 | M | 2007 | Vertical | ART +ve | 804 |
| VT5 | 38 | F | 2006 | Heterosexual | ART –ve | 96 |
| VT6 | 6 | M | 2006 | Vertical | ART –ve | 1048 |
| VTD8 | 27 | F | 2006 | Heterosexual | ART –ve | 475 |
| VTE8 | 5 | F | 2006 | Vertical | ART +ve | 870 |
| D1 | 30 | F | 2008 | Heterosexual | ART –ve | 419 |
| E1 | 9 | M | 2008 | Vertical | ART +ve | NA |
| D2 | 30 | F | 2008 | Heterosexual | ART +ve | 403 |
| E2 | 6 | M | 2008 | Vertical | ART –ve | 972 |
| D19 | 30 | F | 2008 | Heterosexual | ART +ve | 310 |
| E19 | 10 | M | 2008 | Vertical | ART +ve | 720 |
| D43 | 31 | F | 2008 | Heterosexual | ART +ve | 430 |
| E43 | 8 | M | 2008 | Vertical | ART –ve | 734 |
| D47 | 28 | F | 2008 | Heterosexual | ART +ve | 390 |
| E47 | 7 | M | 2008 | Vertical | ART +ve | 732 |
| D48 | 27 | F | 2008 | Heterosexual | ART +ve | 310 |
| E48 | 8 | M | 2008 | Vertical | ART +ve | 720 |
| D63 | 30 | F | 2008 | Heterosexual | ART –ve | 212 |
| E63 | 10 | M | 2008 | Vertical | ART +ve | 458 |
| D64 | 35 | F | 2008 | Heterosexual | ART +ve | 403 |
| E64 | 7 | M | 2008 | Vertical | ART +ve | 572 |
| A4 | 30 | M | 2004 | Heterosexual | ART –ve | 351 |
| A5 | 28 | F | 2008 | Heterosexual | ART –ve | 972 |
| A6 | 28 | F | 2006 | Heterosexual | ART –ve | 1046 |
| A7 | 35 | M | 2008 | Heterosexual | ART –ve | 519 |
| A8 | 39 | M | 2006 | Heterosexual | ART –ve | 447 |
| A9 | 35 | M | 2006 | Heterosexual | ART –ve | 440 |
| N11 | 32 | M | 2010 | Heterosexual | ART +ve | 140 |
| N12 | 27 | F | 2010 | Heterosexual | ART +ve | 226 |
| N13 | 39 | F | 2010 | Heterosexual | ART +ve | 104 |
| N14 | 40 | M | 2010 | Heterosexual | ART +ve | 198 |
| N15 | 50 | M | 2010 | Heterosexual | ART +ve | 74 |
| N16 | 30 | M | 2010 | Heterosexual | ART +ve | 256 |
| N17 | 40 | M | 2010 | Heterosexual | ART +ve | 114 |
| N45 | 30 | M | 2010 | Heterosexual | ART –ve | 153 |
| N46 | 25 | M | 2010 | Heterosexual | ART –ve | 125 |
| N47 | 35 | F | 2010 | Heterosexual | ART –ve | 339 |
| N48 | 24 | F | 2010 | Heterosexual | ART –ve | 400 |
|  |  |  |  |  |  |  |
| NOTE: |  |  |  |  |  |  |
| NA - Not Available | |  |  |  |  |  |
| ART –ve - naïve | |  |  |  |  |  |
| ART +ve - positive | |  |  |  |  |  |
| Letter D denotes mother and E denotes child | | | |  |  |  |

Table S1. **Clinical data for HIV-1 infected patients from North India (n=72)**

| Mutations | HIV-1 negative | | HIV-1 positive | |  |  |
| --- | --- | --- | --- | --- | --- | --- |
|  | Total number of individuals carrying the mutation | Total individuals (n) | Total number of individuals carrying the mutation | Total individuals (n) | p value | q value (post Benjamini-Hochberg multiple testing correction) |
| K26R* | 1 | 70 | 0 | 72 | 0.3088 | 0.3143 |
| L55Q* | 3 | 70 | 0 | 72 | 0.0758 | 0.2058 |
| F166L* | 1 | 70 | 0 | 72 | 0.3088 | 0.3143 |
| Δ32* | 2 | 70 | 0 | 72 | 0.1486 | 0.2124 |
| **Q194H*** | **4** | **70** | **0** | **72** | **0.0396** | **0.1727** |
| R223Q* | 2 | 70 | 0 | 72 | 0.1486 | 0.2124 |
| Δ228 K* | 1 | 70 | 0 | 72 | 0.3088 | 0.3143 |
| I253T* | 1 | 70 | 0 | 72 | 0.3088 | 0.3143 |
| F299S* | 1 | 70 | 0 | 72 | 0.3088 | 0.3143 |
| **R319H*** | **4** | **70** | **0** | **72** | **0.0396** | **0.1727** |
| I12V* | 1 | 70 | 0 | 72 | 0.3088 | 0.3143 |
| C20R* | 2 | 70 | 0 | 72 | 0.1486 | 0.2124 |
| **L55P*** | **4** | **70** | **0** | **72** | **0.0396** | **0.1727** |
| R60K* | 1 | 70 | 0 | 72 | 0.3088 | 0.3143 |
| R60G* | 1 | 70 | 0 | 72 | 0.3088 | 0.3143 |
| C101Y* | 2 | 70 | 0 | 72 | 0.1486 | 0.2124 |
| F118L* | 1 | 70 | 0 | 72 | 0.3088 | 0.3143 |
| F118S* | 1 | 70 | 0 | 72 | 0.3088 | 0.3143 |
| W153L* | 3 | 70 | 0 | 72 | 0.0758 | 0.2058 |
| S215P* | 1 | 70 | 0 | 72 | 0.3088 | 0.3143 |
| R225P* | 1 | 70 | 0 | 72 | 0.3088 | 0.3143 |
| E330K* | 3 | 70 | 0 | 72 | 0.0758 | 0.2058 |
| S336G* | 1 | 70 | 0 | 72 | 0.3088 | 0.3143 |
| S17P* | 2 | 70 | 0 | 72 | 0.1486 | 0.2124 |
| V25A* | 2 | 70 | 0 | 72 | 0.1486 | 0.2124 |
| **S38L*** | **4** | **70** | **0** | **72** | **0.0396** | **0.1727** |
| L50P* | 3 | 70 | 0 | 72 | 0.0758 | 0.2058 |
| V51A* | 2 | 70 | 0 | 72 | 0.1486 | 0.2124 |
| L77P* | 2 | 70 | 0 | 72 | 0.1486 | 0.2124 |
| L81P* | 2 | 70 | 0 | 72 | 0.1486 | 0.2124 |
| F85L* | 3 | 70 | 0 | 72 | 0.0758 | 0.2058 |
| F112L* | 2 | 70 | 0 | 72 | 0.1486 | 0.2124 |
| F158L* | 2 | 70 | 0 | 72 | 0.1486 | 0.2124 |
| F158V* | 2 | 70 | 0 | 72 | 0.1486 | 0.2124 |
| **H181S*** | **4** | **70** | **0** | **72** | **0.0396** | **0.1727** |
| I198V* | 2 | 70 | 0 | 72 | 0.1486 | 0.2124 |
| I212V* | 2 | 70 | 0 | 72 | 0.1486 | 0.2124 |
| F311S* | 3 | 70 | 0 | 72 | 0.0758 | 0.2058 |
| G344R* | 2 | 70 | 0 | 72 | 0.1486 | 0.2124 |
| **FS at 156*** | **4** | **70** | **0** | **72** | **0.0396** | **0.1727** |
| FS at 166* | 2 | 70 | 0 | 72 | 0.1486 | 0.2124 |
| **FS at 220*** | **4** | **70** | **0** | **72** | **0.0396** | **0.1727** |
| **F107L*** | **0** | **70** | **6** | **72** | **0.0136** | **0.1727** |
| A29T* | 0 | 70 | 2 | 72 | 0.1602 | 0.2124 |
| **A73P*** | **0** | **70** | **4** | **72** | **0.0455** | **0.1727** |
| **W86G*** | **0** | **70** | **6** | **72** | **0.0136** | **0.1727** |
| C101R* | 0 | 70 | 1 | 72 | 0.3224 | 0.3224 |
| **W153R*** | **0** | **70** | **4** | **72** | **0.0455** | **0.1727** |
| R31G* | 0 | 70 | 2 | 72 | 0.1602 | 0.2124 |
| **F41L*** | **0** | **70** | **4** | **72** | **0.0455** | **0.1727** |
| A87V* | 0 | 70 | 2 | 72 | 0.1602 | 0.2124 |
| **R88H*** | **0** | **70** | **4** | **72** | **0.0455** | **0.1727** |
| **A90P*** | **0** | **70** | **5** | **72** | **0.0248** | **0.1727** |
| **G97E*** | **0** | **70** | **4** | **72** | **0.0455** | **0.1727** |
| T177I* | 0 | 70 | 2 | 72 | 0.1602 | 0.2124 |
| R235W* | 0 | 70 | 2 | 72 | 0.1602 | 0.2124 |
| T282A* | 0 | 70 | 2 | 72 | 0.1602 | 0.2124 |

Table S2. **List of all mutations observed in this study with p and q values**

Note: mutations in bold are statistically significant mutations after the Benjamini-Hochberg multiple testing correction
